# Analysis of liver function test abnormalities in kidney transplant recipients: 7 year experience

**DOI:** 10.12669/pjms.326.10725

**Published:** 2016

**Authors:** Oguzhan Sitki Dizdar, Alparslan Ersoy, Savas Aksoy, Banu Demet Ozel Coskun, Abdulmecit Yildiz

**Affiliations:** 1 Oguzhan Sitki Dizdar, Department of Internal Medicine and Clinical Nutrition, Kayseri Training and Research Hospital, Turkey; 2 Alparslan Ersoy, Professor, Department of Internal Medicine, Division of Nephrology, Uludag University Medical School, Turkey; 3 Savas Aksoy, Department of Internal Medicine, Division of Nephrology, Uludag University Medical School, Turkey; 4 Banu Demet Ozel Coskun, Department of Internal Medicine, Division of Gastroenterology, Kayseri Training and Research Hospital, Turkey; 5 Abdulmecit Yildiz, Department of Internal Medicine, Division of Nephrology, Uludag University Medical School, Turkey

**Keywords:** Complication, Cytomegalovirus, Hepatotoxicity, Mycophenolatemofetil, Kidney transplantation

## Abstract

**Objective:**

Immunosuppressive drugs, antimicrobial agents and infectious complications may cause liver function test abnormalities (LFTA) in kidney transplant recipients (KTR). The objectives of this study were to identify the outcome of (LFTA). To identify the risk factors affecting development and severity of hepatotoxicity in KTR.

**Methods:**

We retrospectively evaluated the medical records of KTR. Hepatotoxicity attacks were defined as impairment in liver function tests that was responsive to drug dose reduction or discontinuation, or treatment of specific causes such as infectious complications.

**Results:**

One hundred-fifty-six episodes of hepatotoxicity occurred in 107 patients in 281 KTR, with an incidence of 38%. Patients with hepatotoxicity episodes had a high total mortality rate, higher incidence of positive pre-transplant cytomegalovirus (CMV) IgM test, higher creatinine values during the first month post-transplant, underwent additional acute rejection episodes, and received fewer cyclosporin A based ID. Only positive CMV IgM testing was identified as a significant independent risk factor for hepatotoxicity in our multiple analysis. Mycophenolatemofetil (MMF) related hepatotoxicity was the most common cause of drug related LFTA.

**Conclusions:**

Patients with LFTA can have significant complications. Pre-transplant positive CMV IgM tests predispose transplant recipients to the development of LFTA during the post-transplant period. MMF can be a serious hepatotoxic drug.

## INTRODUCTION

The principle challenge in kidney transplantation is the suppression of allograft rejection. Thus, use of immunosuppressive drugs (ID) is inevitable. ID can elicit a variety of adverse effects, ranging from infection to gastrointestinal and hepatic toxicity.^1^,^2^ Furthermore, during the first six months after transplantation antimicrobial agents are given prophylactically to all kidney transplant recipients (KTR). ID and antimicrobial agents may cause either direct or idiosyncratic hepatotoxicity. In addition to multiple drug usage in KTR, infectious and septic complications make patients vulnerable to liver injury. Induced liver injury is largely a challenging diagnosis of exclusion. There is no gold standard and no specific serum biomarker or characteristic histologic feature that reliably identifies a drug as the cause of toxicity. The diagnosis is especially difficult when affected persons are taking multiple drugs, any one of which might be responsible for hepatotoxicity or might act synergistically with other drugs.^3^-^5^

A population-based survey in the United States conducted between 1999 and 2002 estimated that an abnormal ALT was present in 8.9 percent of respondents.^6^ But there is no research on liver function test abnormalities (LFTA) in KTR population. Although clinical judgment is a necessary first step in the identification of any adverse drug event, this frequently leads to inaccurate reports of hepatic adverse drug reactions.^7^ These considerations have revealed the need to do new research to improve the reliability of causality assessment in cases of hepatotoxicity.^8^

The objective of this study was to identify the characteristics and consequences of LFTA and the risk factors affecting the development and severity of hepatotoxicity in KTR.

## METHODS

In the present observational study, we retrospectively evaluated the medical records of adult recipients who underwent kidney transplant from January 2006 to March 2013 at a teaching hospital and tertiary referral center. This study was approved by the Ethics Committee of Uludag University Medical School. All patients had normal serum bilirubin, serum aspartate aminotransferase (AST), serum alanine aminotransferase (ALT), lactic acid dehydrogenase (LDH), and alkaline phosphatase (ALP) prior to transplantation. The following variables were assessed in all KTR: age, gender, cause of end-stage renal disease, serology for cytomegalovirus (CMV), hepatitis B and hepatitis C status prior to transplantation, dialysis type and duration, donor type (cadaveric or living), history of delayed graft function, acute or chronic rejection, first post-transplant month creatinine levels, and initial immunosuppressive regimen. Hepatotoxicity attacks were defined as impairment in liver function tests that responds to drug dose reduction or discontinuation, or treatment of a specific cause such as infection.

The clinical records of kidney recipients with hepatotoxicity were carefully reviewed, including clinical signs and symptoms, kidney function, laboratory tests, serology, blood and other cultures, radiologic findings, diagnostic and therapeutic procedures, complications during hepatotoxicity, the dose of immunosuppressive and other medications at the time of diagnosis of hepatotoxicity, and the patient response to specific treatment.

All patients received initial immunosuppressive therapy with prednisolone (P), antimetabolites (mycophenolatemofetil-(MMF), azathioprine-(AZA)) combined with calcineurin inhibitors (CNI; cyclosporin A-CyA/, tacrolimus-Tac), interleukin-2 receptor antagonists (IL-2ra) or mechanistic target of rapamycin (mTOR) inhibitors (sirolimus(SRL)/everolimus(EVL)).

All statistical analyses were conducted using SPSS statistical package (version 13.0; SPSS, Chicago, IL, USA). Data are presented as absolute and percentage frequency and mean with standard deviation. The normality and the homogeneity of the data were evaluated by Shapiro-Wilk test and Levene test, respectively. Comparisons between groups for continuous variables were performed using the Student *t* test (normal distribution) or the Mann-Whitney U test (non-normal distribution). Categoric variables were compared using the chi-square test or Fisher exact test when appropriate. We also calculated the relative risk of hepatotoxicity after transplantation using logistic regression. Only the variables with a statistically significant association in the simple logistic regression model were included in the multiple logistic regression model. P < 0.05 was considered statistically significant.

## RESULTS

Of the 281 renal transplant patients, 56% were male and the overall mean age was 35.9± 12.1 years. One hundred-fifty-six episodes of hepatotoxicity occurred in 107 patients following 281 renal transplants, an overall incidence of 38%. Twenty-nine patients experienced two episodes of hepatotoxicity and 10 patients experienced three episodes of hepatotoxicity.

Patients with hepatotoxicity had a high total mortality rate (14% vs. 6.3%) and higher incidence of positive pre-transplant CMV Ig M (15.2% vs 3.6%), relative to patients who did not experience hepatotoxicity (Table-I). We evaluated all statistically significant hepatotoxicity risk factors using multiple regression analysis. Only the presence of a positive pre-transplant CMV IgM test (OR 16.86, 95% CI 1.82 -155.8; p=0.013) was identified as an independent risk factor for hepatotoxicity in the multiple regression analysis. However, use of the CyA/MMF/P treatment was associated with reduced risk of hepatotoxicity (OR 0.32, 95% CI 0.127 – 0.83; p=0.02).

**Table-I:** Characteristics of patients who had hepatotoxicity and others.

Variables	Hepatotoxicity group (107)	Non- hepatotoxicity group (174)	p value
Sex, male/female, n(%)	56(52.3)/51(47.7)	101(58)/73(42)	NS
Male/female ratio	1.09	1.38	NS
Age, in years	36.9 ± 11.6	35.3 ± 12.4	NS
Donor type, cadaveric/living, n(%)	48(44.9)/59(55.1)	72(41.4)/102(58.6)	NS
Dialysis type, HD/PD/Preemptive	79/22/6	113/40/21	NS
Dialysis duration, year	5.2 ± 4.2	5.2 ± 4.0	NS
Initial immunosupressive protocol			
Tac/MMF/P, n(%)	46 (43)	65 (37.4)	NS
CyA/MMF/P, n(%)	32 (29.9)	73 (42)	0.016
EVL/MMF/P, n(%)	24 (22.4)	15 (8.6)	0.002
Other protocols, n(%)	5 (4.7)	21 (12)	NS
Anti HCV, n(%)	0 (0)	4 (2.3)	NS
HBsAG, n(%)	1 (0.9)	3 (1.7)	NS
CMV Ig M, n(%)*	7 (6.5)	3 (1.7)	0.033
Chronic rejection, n(%)	3 (2.8)	2 (1.2)	NS
Acute rejection, n(%)	15 (14)	9 (5.2)	0.04
Delayed graft function, n(%)	38 (35.5)	38 (21.8)	NS
First month creatinin value, mg/dL	1.46 ± 0.47	1.34 ± 0.72	0.013
Mortality, n(%)	15 (14)	11 (6.3)	0.031

HD: Hemodialysis, PD: Peritoneal dialysis, Tac: Tacrolimus, MMF: Mycophenolat mophetil, P: Prednisolone, CyA: Cyclosporin A, EVL: Everolimus, CMV: Cytomegalovirus, NS: Not significant

* Positive test in pretransplant assessment;

All heptotoxicity attacks were classified into three groups according to ALT levels: study group I with ALT levels between the upper limit of normal (ULN) to ULN x 3, study group II with ALT levels of > 3 to 5 times more than the ULN, study group III with ALT level >5 times more than the ULN. The most common cause of liver injury was drugs in all three groups (Table II and III). In 3 patients, more than one drug was responsible for hepatotoxicity. Use of drugs was significantly lower in group 1 relative to the other two groups. Unknown etiology was significantly less prevalent among group 3. The mean time of hepatotoxic attack onset was 5.3 ± 9.2 months (range 1 - 63 month) after transplantation. However, there was no significant difference in the time to hepatotoxicity onset between groups. Mean attack duration was 67.5 ± 94.8 days (range 2 – 735) for remitting attacks. A total of 17 attacks remained floating course or not remitted. Attack duration was significantly shorter in group 1 in the other two groups (53.9 ± 99.9; 92.4 ± 67.7; 77.7 ± 100.6, group1; 2; 3, respectively).

**Table-II:** Laboratory findings of hepatotoxicity attacks.

Variable	Hepatotoxicity attacks (156)
AST, U/L	125 ± 235
ALT, U/L	200 ± 253
Creatinine, mg/dL	1.98 ± 1.49
ALP, U/L	108 ± 86
GGT, U/L	165 ± 228
Urea, mg/dL	84.4 ± 53.9
Total bilirubin, mg/dL	0.92 ± 1.41
Direct Bilirubin, mg/dL	0.53 ± 0.95
LDH, U/L	288 ± 106

AST: aspartate aminotransferase, ALT: alanine aminotransferase, GGT: gamma glutamyl transferase, ALP: alkaline phosphatase, LDH: Lactate dehydrogenase.

**Table-III:** Etiologic differences between three groups.

Etiology	All hepatotoxicity attacks(156)	Group1(83)	Group 2(34)	Group 3 (36)	p value
Drugs, n(%)*	68 (43.6)	27 (32.1)	18 (51.4)	23 (62.2)	0.005
MMF, n(%)	26 (16.6)	9 (10.8)	6 (17.6)	11 (30.5)	0.035
Tac, n(%)	13 (8.3)	5 (6)	4 (11.4)	4 (10.8)	-
CyA, n(%)	4 (2.6)	2 (2.4)	0 (0)	2 (5.4)	-
EVL, n(%)	1 (0.6)	0 (0)	1 (2.9)	0 (0)	-
Sirolimus, n(%)	1 (0.6)	0 (0)	0 (0)	1 (2.7)	-
Antibiotics, n(%)	26 (16.6)	12 (14.3)	7 (20)	7 (18.9)	NS
TMP/SMX, n(%)**	10 (6.4)	5 (6)	2 (5.7)	3 (8.1)	-
Unknown etiology, n(%)***	63 (40.4)	43 (51.2)	15 (42.9)	5 (13.5)	<0.001
Sepsis/hypoxy, n(%)	8 (5.1)	4 (4.8)	2 (5.7)	2 (5.4)	NS
CMV, n(%)	8 (5.1)	2 (2.4)	0 (0)	6 (16.2)	-
Hyperlipidemia, n(%)	4 (2.6)	4 (4.8)	0 (0)	0 (0)	-
Hepatitis B, n(%)	2 (1.3)	2 (2.4)	0 (0)	0 (0)	-
Acute pancreatitis, n(%)	1 (0.6)	1 (1.2)	0 (0)	0 (0)	-
Cholelithiasis, n(%)	1 (0.6)	0 (0)	0 (0)	1 (2.7)	-
Drug + CMV, n(%)	1 (0.6)	1 (1.2)	0 (0)	0 (0)	-

Tac: Tacrolimus, MMF: Mycophenolat mophetil, P: Prednisolone, CyA: Cyclosporin A, EVL: Everolimus, TMP/SMX: trimethoprim/sulfamethoxazole, CMV: Cytomegalovirus

* Significant differences were available between group 1-2 and group1-3;

** We evaluated the TMP/SMX as an antibiotic, and we made statistical analysis for TMP/SMX according to this.

*** Significant differences were available between group 1-3 and group 2-3. NS: Not significant.

## DISCUSSION

Liver injury is prevalent in this cohort of KTR undergoing a variety of treatment regimes. Liver function test abnormalities in KTR have not been previously investigated in detail. The present study is the first to define details of hepatotoxicity in KTR. We conducted an extensive investigation of LFTA in KTR. Patients with hepatotoxicity had higher total mortality rate and underwent more acute rejection episodes, this finding showed the importance of this issue.

LFTA occurred in 107 (38%) of 281 kidney transplant recipients. Klintmalm et al. reported 19.7% LFTA in 66 recipients of cadaveric kidneys treated with cyclosporin A and prednisone.^9^ Our study found a higher incidence of hepatotoxicity, but this may be related to drugs other than calcineurin inhibitors. In the present study, mean attack duration was 67.5 ± 94.8 days (range 2 – 735). In the study by Balal et al., the median time to normalization of liver function was 16 (4-210) days; this is shorter than the normalization time observed in our study.^10^ The study by Balal et al. assessed only MMF-related LFTA, while the present study assessed all causes of LFTA. In our study, 17 cases of hepatotoxicity floating course or not remitted.

High dose calcineurin inhibitors frequently cause mild elevation of liver tests. Although rare, severe hepatotoxicity may occur. We found 13 cases of hepatotoxicity caused by tacrolimus and four cases of hepatotoxicity resulting from cyclosporine. Hepatotoxicity sometimes makes it necessary to switch between these two drugs. In most reports, tacrolimus hepatotoxicity has been characterized by elevated levels of hepatocellular enzymes, either alone or with minimal cholestasis and hyperbilirubinemia. Ganchow et al. have reported tacrolimus induced cholestatic syndrome following pediatric liver transplantation.^11^ Yadav et al. reported a case of tacrolimus-induced hepatotoxicity in the form of cholestatic hepatitis in a renal transplant recipient whose hepatotoxicity did not decrease after dose reduction; however, normalization of liver enzymes occurred after discontinuing tacrolimus.^12^ Cyclosporine hepatotoxicity has also been reported to cause cholestasis^9^,^13^, but reduction of the cyclosporine dosage alone was sufficient to resolve the presumed hepatotoxicity.^13^ Taniai et al presented a case in which hepatotoxicity was induced by both tacrolimus and cyclosporine after living donor liver transplantation.^14^ A case presented by Mesar et al. reported complete resolution of LFTA after withdrawal of calcineurin inhibitor (tacrolimus) and replacement with sirolimus.^15^ It is important to be aware of the possible association of tacrolimus with hepatotoxicity in order to discontinue therapy and replace with sirolimus in cases of hepatotoxicity.

Hepatotoxicity was a minor problem associated with the use of cyclosporine A in one previous study.^9^ We observed 4 cases of hepatotoxicity associated with cyclosporin A, two of which were mild. Our analysis of hepatotoxicity risk factors found that LFTA risk was decreased in KTR who received an initial immunosuppressive protocol that included CyA/MMF/P. This situation needs further investigation.

In clinical trials, elevated aminotransferase is more commonly associated with SRL rather than CyA treatments or SRL + CyA treatments.^16^-^18^ Franco-Esteve et al reported only a single incidence of hepatotoxicity among 47 patients treated with mTOR inhibitor monotherapy and Jacques et al reported severe sirolimus-inducedacutehepatitis.^19^,^20^ Interestingly sirolimus was a rescue therapy in one KTR who experienced tacrolimus-related hepatotoxicity.^15^ We found only one case of sirolimus related hepatotoxicity. Sirolimus was not included among our initial immunosupressive drugs and was used in only a few patients.

The most common adverse effects of MMF are gastrointestinal and hematological^1^. Nephrotoxicity and overt hepatotoxicity have not been reported. In Balal et al.’s study, MMF-related hepatotoxicity was assessed in renal transplant recipients.^10^ Among the 79 patients, 11 patients (13.9%) exhibited a progressive increase in liver enzymes. High liver enzyme levels regressed after withdrawal (n=6) or reduced dosage (n=5) of MMF. Contrary to expectation, in the present study MMF related hepatotoxicity was the most common cause of drug related LFTA and 26 (16.6%) patients had MMF related hepatotoxicity, with 11 of these cases involving serious LFTA. Balal et al.’s study and our study demonstrate that MMF can be a common cause of drug related hepatotoxicity.^10^ This is a potentially important finding. The side effect profile of drugs may be specific to the study population. In contrast to cyclosporine and tacrolimus, the serum concentration of MMF has not been measured. As a results, it is difficult to predict adverse effects of MMF.

LFTA induced by anaesthetics during the perioperative period is considered as a significant problem. Some reports describe cases of lethal hepatic failure in patients undergoing kidney transplantation after anaesthesia^21^, however of the 7-year period of our study we found no cases of anaesthetic-related LFTA.

Other important etiologies of LFTA include sepsis/hypoxy and CMV infection. Infection is a well known cause of LFTA. Only positive CMV IgM testing was identified as a significant independent risk factor for hepatotoxicity in our multipl analysis. Furthermore, 6 of 8 cases of CMV-induced LFTA were more severe. CMV infection is an important risk factor for the development of LFTA in KTR.

One of the limitations of our study is that combination of immunosuppressive agents in several groups makes it inappropriate to attribute hepatotoxicity to MMF or tacrolimus individually as cumulative hepatotoxicity can occur. Secondly, there is a large cohort of patients where the etiology of hepatotoxicity is unaccounted for.

Consequently, the nature and cause of LFTA must be accurately determined to maximize the benefits and minimize the morbidity associated with immunosupressants or other drugs.

## CONCLUSION

MMF can be a serious hepatotoxic drug. Failure to quickly address MMF-related hepatotoxicity can result in the need for long-term therapy. Secondly, positive pre-transplant CMV IgM test results are associated with a high risk of LFTA during the post-transplant recovery.
